# Genotypic variation in transpiration efficiency due to differences in photosynthetic capacity among sugarcane-related clones

**DOI:** 10.1093/jxb/erx107

**Published:** 2017-04-22

**Authors:** Chunjia Li, Phillip Jackson, Xin Lu, Chaohua Xu, Qing Cai, Jayapathi Basnayake, Prakash Lakshmanan, Oula Ghannoum, Yuanhong Fan

**Affiliations:** 1 Yunnan Sugarcane Research Institute, Yunnan Academy of Agricultural Sciences, 363 Eastern Linquan Road, Kaiyuan 661600, Yunnan Province, P.R. China; 2 CSIRO, Australian Tropical Science Innovation Precinct, Private Mail Bag PO, Aitkenvale, QLD 4814, Australia; 3 Biotechnology and Germplasm Resources Institute, Yunnan Academy of Agricultural Sciences, 9 Xueyun Road, Kunming 650221, Yunnan Province, P.R. China; 4 Sugar Research Australia Limited, PO Box 117, Ayr, QLD 4807, Australia; 5 Sugar Research Australia Limited, 50 Meiers Road, Indooroopilly, QLD 4068, Australia; 6 ARC Centre of Excellence for Translational Photosynthesis, Hawkesbury Institute for the Environment, Western Sydney University, Locked Bag 1797, Penrith NSW 1797, Australia

**Keywords:** Breeding, genotypic variation, photosynthesis, sugarcane, transpiration efficiency.

## Abstract

Sugarcane, derived from the hybridization of *Saccharum officinarum*×*Saccharum spontaneum*, is a vegetative crop in which the final yield is highly driven by culm biomass production. Cane yield under irrigated or rain-fed conditions could be improved by developing genotypes with leaves that have high intrinsic transpiration efficiency, TE_i_ (CO_2_ assimilation/stomatal conductance), provided this is not offset by negative impacts from reduced conductance and growth rates. This study was conducted to partition genotypic variation in TE_i_ among a sample of diverse clones from the Chinese collection of sugarcane-related germplasm into that due to variation in stomatal conductance versus that due to variation in photosynthetic capacity. A secondary goal was to define protocols for optimized larger-scale screening of germplasm collections. Genotypic variation in TE_i_ was attributed to significant variation in both stomatal and photosynthetic components. A number of genotypes were found to possess high TE_i_ as a result of high photosynthetic capacity. This trait combination is expected to be of significant breeding value. It was determined that a small number of observations (16) is sufficient for efficiently screening TE_i_ in larger populations of sugarcane genotypes The research methodology and results reported are encouraging in supporting a larger-scale screening and introgression of high transpiration efficiency in sugarcane breeding. However, further research is required to quantify narrow sense heritability as well as the leaf-to-field translational potential of genotypic variation in transpiration efficiency-related traits observed in this study.

## Highlight

Genotypic variation in both average stomatal conductance operating levels and photosynthesis rates for given conductance levels make important contributions to the variation in intrinsic transpiration efficiency in diverse sugarcane-related germplasm.

## Introduction

Sugarcane is a major tropical and subtropical crop derived from interspecific hybridization of *Saccharum officinarum*×*Saccharum spontaneum* (Roach, 1989). Sugarcane is harvested worldwide in a greater quantity than any other crop, with about 1.9 billion tonnes harvested in 2014 (FAO, 2015). It is the main source of sugar production worldwide and increasingly important in bio-energy production (Cardona *et al.*, 2010). Production areas of sugarcane mostly lie in rain-fed regions, but there is also a significant area that is irrigated. Water stress is a major limitation to growth in many rain-fed production regions (Passioura and Angus, 2010). Given that irrigation costs and competing demands for water in sugarcane production regions are predicted to grow, the development of sugarcane cultivars with enhanced water-use efficiency is becoming increasingly desirable.

Crop yield in any water-limited environment can be expressed in terms of the following components:

$$\begin{array}{l} \text{Yield}=\text{water transpired}\times \text{transpiration efficiency }(\text{TE})\\ \;\times \text{ harvest index} \end{array}$$ (1)

This expression has been useful for researchers to conceptualize and interpret causes of variation in yield in water-limited environments (e.g. Passioura, 1977; Condon and Richards, 1993; Richards *et al.*, 2002). In sugarcane, harvest index can be defined as the proportion of above-ground biomass present as millable cane, and typically is approximately 0.8 at harvest (Robertson *et al.*, 1996). Genotypic improvement for biomass yield can potentially arise only through greater water use (transpiration), or greater biomass per amount of water transpired (TE). In environments where available soil water is used prior to harvest, increases in yield through genetic improvements can arise only by improving water-use efficiency or harvest index (Ghannoum, 2016).

Leaf CO_2_ assimilation (*A*) and H_2_O vapour transpiration (*E*) rates can be expressed as:

$$A=g_{s\_CO_{2}}(Ca-Ci)$$ (2)

and

$$E=g_{s\_H_{2}O}(ei-ea)$$ (3)

where C_i_ and C_a_ are the leaf intercellular and ambient CO_2_ partial pressures, and e_i_ and e_a_ the H_2_O vapour pressures inside the leaf and in the surrounding air, respectively. In addition, g_s*_*H2O_ = 1.6g_s_CO2_, where g_s_CO2_ and g_s_H2O_ refer to the stomatal conductance (g_s_) for CO_2_ and H_2_O vapour, respectively, and 1.6 is the ratio of binary diffusivity of H_2_O vapour to that of CO_2_ in air (Farquhar *et al.*, 1989). Accordingly, leaf-level transpiration efficiency (TE_L_) is given by:

$$TE_{L}=\frac{A}{E}=\frac{C_{a}\;(1-C_{i}/C_{a})}{1.6(e_{i}-e_{a})}$$ (4)

Whereas assimilation rates depend on the CO_2_ supply (stomatal conductance) and demand (photosynthetic capacity) functions, transpiration rates depend on stomatal and boundary layer conductance as well as the leaf-to-air vapour pressure difference, which in turn depends on leaf temperature and the relative humidity of the surrounding air. Another expression that excludes the direct effects of vapour pressure in surrounding air and is commonly used in comparing genotypes is termed intrinsic transpiration efficiency, TE_i_, given by (Farquhar *et al.*, 1989):

$$TE_{i}=\frac{A}{g_{s}}=1-\frac{C_{i}}{C_{a}}$$ (5)

Reduced g_s_ leads to lower C_i_ and C_i_/C_a_, which represents an integrative parameter of TE_i_, reflecting changes in both *A* and g_s_ (equation 5).

Recently, Jackson *et al.* (2016) reported significant genetic variations in leaf and whole-plant TE in sugarcane and related germplasm, and observed that C_i_ had a negative genotypic correlation with whole-plant TE, at least at mid-range g_s_ levels.

While there is usually a close positive relationship between *A* and g_s_, it has been shown in many species that this relationship is not linear, such that the slope of *A* versus g_s_ decreases as g_s_ becomes larger (Gilbert *et al.*, 2011). Thus, in examining variation within any population of genotypes, relatively high TE_i_ in any particular genotype may arise because of low conductance, or because of greater photosynthetic capacity compared with other genotypes for a given conductance (Gilbert *et al.*, 2011). In addition, while enhancing TE may be a worthwhile goal in crop improvement programmes, a potential problem arises because a negative covariance is frequently observed between transpiration and TE (Condon *et al.*, 2004; Blum, 2005, 2009; Sinclair, 2012; Vadez *et al.*, 2014). For example, if high TE_i_ arises mainly due to reduced g_s_, this may be associated with reduced water use and productivity. By contrast, increased TE_i_ due to a higher rate of photosynthesis at any given level of conductance (and leading to lower *C*_i_) may be expected to be of more general agronomic value (Ghannoum, 2016).

Sugarcane is a crop in which the final yield is highly driven by total biomass production, unlike most grain crops, for which environmental conditions impacting the timing of flowering and grain filling complicate the relationship between TE, water use, and crop yield. Hence, in sugarcane, photosynthetic capacity is likely to be more directly related to crop yield than is the case with grain crops.

The aim of this study was to partition genotypic variation in leaf-level TE_i_ in a sample of diverse sugarcane-related genotypes into that attributable to the variation in photosynthetic capacity (TEpc) and that attributable to the variation in stomatal conductance (TEg_s_), following the concepts advocated by Gilbert *et al.* (2011). The genotypes examined were sampled from the extensive Chinese sugarcane-related germplasm collection. The genera and species contained in this collection are from the so-called *Saccharum* complex (Mukherjee, 1957), which are believed to be involved in the evolution of sugarcane and are able to be crossed with sugarcane (Berding and Roach, 1987). Previous genetic diversity studies using DNA markers indicated some relationships between and within species in this collection (Cai *et al.*, 2005) and were used to support the sampling methodology in this study. A stratified random sample of genotypes was taken that represents a range of species and a major portion of the genotypic diversity contained within this germplasm collection. The results from this study are discussed in relation to whether an introgression breeding programme targeting improvement of TE_i_ would be of value, and to help define methodology that could be used for efficiently screening larger populations of sugarcane genotypes in any future introgression breeding programmes.

## Materials and methods

### Overview of the experiment and germplasm collection

**Fig. 1.**
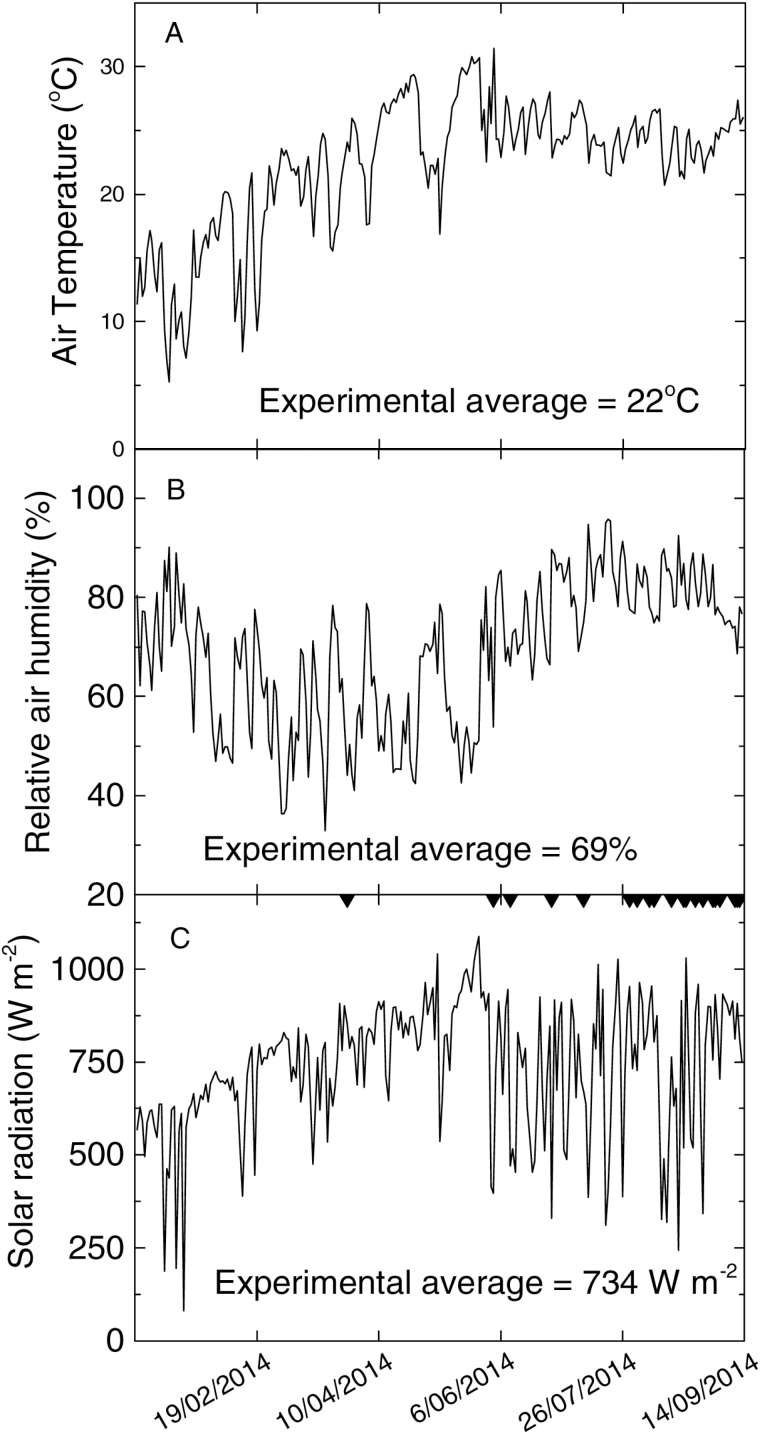
Weather conditions during the experimental period. (**A**) Daily average temperature and (**B**) humidity, and (**C**) maximum (on an hourly basis) solar radiation during the experimental period. Plants were transplanted on 21 December 2013 and measured between 28 March 2014 and 12 September 2014. Days of measurement are indicated with tick marks on the top border of panel C.

Twenty clones of sugarcane-related germplasm (Table 1) were grown under well-watered conditions at the National Germplasm Repository of Sugarcane in Kaiyuan (NGRS-KY), located within a commercial sugarcane-growing region in Yunnan Province, Southwestern China (103.25°E, 23.70°N, altitude 1052 m). Weather conditions during the experiment are shown in Fig. 1. The NGRS-KY is the major field gene bank for conservation of sugarcane and related germplasm clones in China. The repository, which was established in 1995 and expanded in 2003, seeks to explore, collect, characterize, and provide materials for use in sugarcane research and breeding programmes, as well as to exchange germplasm with institutes and organizations around the world. Currently, the repository is 2.3 ha in area and contains 2664 accessions across 15 species in five genera. Most of the germplasm clones were domestically collected from 14 provinces in southern China. Foreign clones, received through exchange pathways, are also included (http://www.yngzs.net/news_detailkjcx/newsId=139.html). The 20 clones sampled in this experiment (Table 1) were selected to represent most of the species curated in the collection, including replication of clones from some of the largest species groups. The selected clones included a set of wild species and a number of commercial cultivars selected from breeding programmes and bred for high sugar content and cane yield.

**Table 1.** Summary of leaf gas exchange characteristics for 20 sugarcane-related clones measured in the germplasm screening experiment

Clone	Species	*A* (μmol m^−2^ s^−1^)	g_s_ (mol m^−2^ s^−1^)	C_i_ (μL L^−1^)	TE_i_ (*A*/g_s_) (μmol mol^−1^)
51NG63	*Saccharum robustum*	23.1	0.169	122	142
Guangxi87-20	*Saccharum spontaneum*	20.2	0.146	128	141
Hainanlingshui4	*Saccharum spontaneum*	20.8	0.154	132	138
India2	*Saccharum spontaneum*	24.9	0.185	124	139
Yunnan2009-2	*Saccharum spontaneum*	23.3	0.183	137	132
Uba	*Saccharum sinense*	13.9	0.095	123	150
96NG16	*Saccharum officinarum*	23.9	0.176	124	140
Pansahi	*Saccharum barberi*	16.8	0.13	143	135
Guangdong64^#^	*Narenga porphyrocoma*	17.6	0.127	129	143
Yunnan95-35^#^	*Miscanthus sinensis*	10	0.076	156	133
Guangxi79-8^#^	*Miscanthus floridulus*	10.3	0.07	129	150
Guangdong2010-102^#^	*Imperata cylindrica*	12.6	0.09	133	145
Yunnan95-20^#^	*Erianthus rockii*	15.8	0.112	121	149
Yunnan97-4^#^	*Erianthus fulvus*	12	0.093	159	129
Hainan92-84^#^	*Erianthus arundinaceus*	14.1	0.088	102	164
KQ01-1390	Commercial cultivar^a^	17.9	0.131	134	139
Q208	Commercial cultivar^a^	16.8	0.13	146	133
ROC22	Commercial cultivar^b^	20.4	0.157	142	132
Yuetang93-159	Commercial cultivar^b^	16.8	0.126	136	139
Yunzhe03-194	Commercial cultivar^b^	22.3	0.174	132	136
Mean		17.4	0.128	133	141
Least significant difference (*P* < 0.05)		2.1	0.018	12.3	7.7

The clones were grown outdoors in pots or in the field. Plants were well watered and fertilized. Leaf gas exchange measurements were made at a photosynthetically active radiation of 1200 μmol m^−2^ s^−1^, CO_2_ of 400 µl L^−1^, and under ambient temperature and humidity. Values are the means of four replicates per clone measured over 20 days. The averages of each parameter across all measurements are shown. Commercial cultivars in ^a^Australia and ^b^China refer to a complex derivative of *Saccharum officinarum*×*Saccharum spontaneum*. ^#^Transplanted from rhizomes instead of stem cuttings.

### Experimental design

The experimental design was a randomized complete block design with four blocks. Three blocks comprised plants grown in pots, and one block consisted of plants grown in the ground. Each clone was represented by one pot or plant within each of the four blocks. All plants were grown outdoors under ambient conditions (Fig. 1). Single-bud stem cuttings of each clone were first transplanted into bowls on 21 December 2013 for germination until the three- to four-leaf stage, and then transferred on 15 January 2014 into 22.1 L pots (35 cm height and 50 cm diameter), filled with the local nursery soil, a typical subtropical red earth. For clones which had lateral buds reluctant to sprout, rhizomes were used instead of stem cuttings (Table 1). Two plants per clone were transplanted into each pot. Plants grown in the ground were established similarly to potted ones.

Potted plants were well watered daily, while in-ground plants were flood-irrigated once every month. Calcium superphosphate and urea (LvBao, Kunming, China) were applied as base fertilizer when the seedlings and rhizomes were planted into the pots or in the field. Subsequently, slow-release fertilizer (LvBao) was applied near the stem base every 4–5 weeks. Weeds were removed by hand, and herbicide (Paraquat; Syngenta, Guangzhou, China) was sprayed onto the weeds, avoiding the clones. Granular pesticide (Bisultap, Kunming, China) for insect stem borer control was applied in the same way as the base fertilizer. Liquid pesticide (chlorantraniliprole-thiamethoxam; Syngenta) was sprayed onto the plants 3 days after they were transplanted. Subsequent pesticide applications were employed as required.

### Measurements

Leaf gas exchange measurements were conducted over the course of the experiment using a portable open gas exchange system (LI-6400; Li-Cor, Lincoln, NE, USA) to determine *A*, g_s_, C_i_, the ratio of intercellular to ambient CO_2_ (C_i_/C_a_), and instantaneous leaf-level TE_i_ (*A*/g_s_). Measurements were conducted between 10:00 and 14:00 on attached, last fully-expanded leaves. Measurements were made at a photosynthetic photon flux density of 1200 μmol m^−2^ s^−1^, a CO_2_ concentration of 400 μl L^−1^, and ambient temperature and humidity. Across all measurements, leaf temperature varied between 24 and 42°C, and leaf-to-air vapour pressure deficit ranged between 1 and 6 kPa. Before each measurement, the leaf was allowed to stabilize until it reached a steady state of CO_2_ uptake. Leaf gas exchange was measured for all plants on 20 different days spread between 28 March 2014 and 12 September 2014. On several dates, not all pots were measured, with a total of 1325 measurements being made.

### Partitioning of TE_i_ variations

Genotypic effects for TE_i_ were partitioned into two independent sources of variation: one source attributed to variation in stomatal conductance (TEg_s_) and the other attributed to variation in photosynthetic capacity (TEpc). This partitioning followed the concepts described by Gilbert *et al.* (2011), but with some modifications. Given the general curvilinear relationship between g_s_ and *A* resulting in a negative relationship between TE_i_ and g_s_, on average, genotypes with low g_s_ measures are expected to have higher TE_i_ than genotypes with high g_s_. Hence, the impact of g_s_ on TE_i_ may be due to stomatal closure contributing to lower C_i_ and higher TE_i_, as explained by Condon *et al.* (2002), among others. Accordingly, for each measurement of leaf gas exchange, TEg_s_ was defined as the TE_i_ expected if the photosynthetic capacity was determined from a reference (*A* versus g_s_) function for the measured value of g_s_, and expressed as a deviation from the mean TE_i_ based on all observations. Unlike Gilbert *et al.* (2011), the reference (*A* versus g_s_) function was derived from observations from all genotypes rather than just those from an individual comparator genotype. TEpc for each measurement point was defined as the deviation of the actual observed TE_i_ from the TEg_s_ (i.e. TE_i_ – TEg_s_; the deviation of the observed TE_i_ from the expected population average at the measured conductance level).

### Data analysis

Data were analysed using SAS version 9.4, PROC MIXED, and PROC GLM procedures. All measurements for each trait (*A*, g_s_, C_i_, TE_i_, TEpc, TEg_s_) were pooled for analysis and total variance was partitioned into dates and genotypes main effects, clone × date interaction effects, and residual ‘error’ variation. In the mixed model analyses, dates and genotypes were considered as random effects and variance components estimated accordingly using the restricted maximum likelihood method. *P* values for significance testing of genotype effects were obtained from the PROC GLM procedure using the same partitioning of effects.

**Fig. 2.**
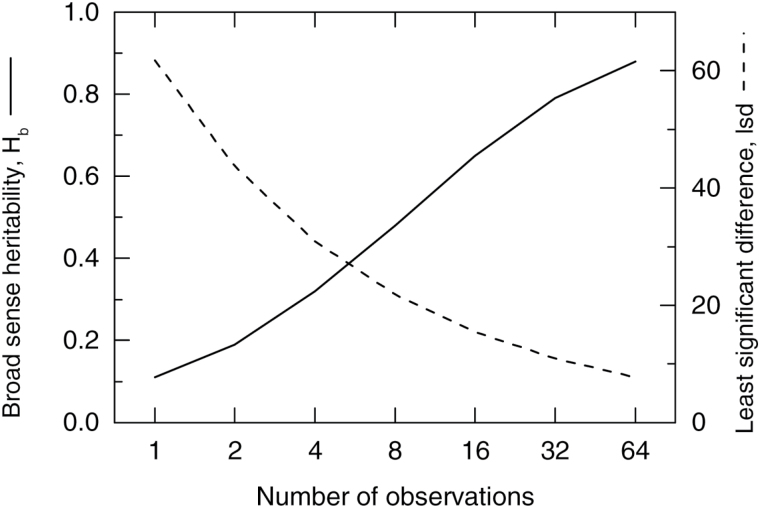
Predicted effect of number of observations on H_b_ and least significant difference.

Broad sense heritability (H_b_) for traits was determined from Falconer and Mackay (1996):

$$H_{b}=\frac{\sigma_{g}2}{\sigma_{p}2}$$ (6)

where σ_g_^2^ and σ_p_^2^ are the genotypic and phenotypic variance, σ_p_^2^ = σ_g_^2^ + σ_g.date_^2^/*d* + σ_e_^2^/*n*, σ_g.date_^2^ = clone × date interaction variance, *d* = number of different dates of measurement, σ_e_^2^ = error variance, and *n* = total number of observations per genotype. For the purpose of estimating H_b_, it was assumed that *n* = 63 (this value in fact ranged from 63 to 70). H_b_ and least significant differences were also determined for TE_i_ for a range of different *n* values (Fig. 2), to simulate expected values if different numbers of observations were made in future experiments involving screening of more germplasm.

The genotypic coefficient of variation (GCV, %), an indicator of relative genotypic variation, was determined for each trait from:

$$\text{GCV}=\frac{\sigma_{\text{g}}}{\bar{\text{x}}}\times \text{100}$$ (7)

where σ_g_ is the standard deviation of the genotypic effect, $\bar{\text{x}}$ is the population mean, and σ_g_^2^ is the genotypic variance. For the measures of TEg_s_ and TEpc where averages are near zero (because these values are expressed as deviations from the average of all observations), the value used for $\bar{\text{x}}$ was that for TEi.

Expected genotypic gain from different combinations of measurement number and number of clones was predicted using the following standard formula for gain from selection (Falconer and Mackay, 1996):

$$\text{Gain}=i\times h\times \sigma_{\text{g}}$$ (8)

where *i* = intensity of selection; obtained from Appendix Table A in the text by Falconer and Mackay (1996), *h* = square root of broad sense heritability, and σ_g_ = genotypic standard deviation.

Pearson’s correlation coefficients among the various parameters were calculated using SPSS 19.0. The correlations among gas exchange parameters including *A*, g_s_, C_i_, and TE_i_ were evaluated for all the data collected in the germplasm experiment.

## Results

### Variation in measurements

Significant genotypic variation was observed for leaf-level *A*, g_s_, *C*_i,_ TE_i_, TEpc, and TEg_s_ among the clones (Tables 1 and 2). A 2.5-fold variation in *A* was observed, with values ranging from an average of 10 μmol m^−2^ s^−1^ in Yunnan95-35 (*Miscanthus sinensis*) to 24.9 μmol m^−2^ s^−1^ in India2 (*Saccharum spontaneum*). Similarly, a 2.6-fold proportional variation was observed for g_s_, with this parameter ranging from an average of 0.07 mol m^−2^ s^−1^ in Guangxi79-8 (*M. floridulus*) to 0.185 mol m^−2^ s^−1^ in India2 (*S. spontaneum*). Smaller, but significant genotypic variations in C_i_ and TE_i_ (1.6-fold and 1.7-fold, respectively) were also observed. Yunnan97-4 (*Erianthus fulvus*) exhibited the highest average C_i_ (159 μL L^−1^) and lowest average TE_i_ (129 μmol CO_2_ mol^−1^ H_2_O), while Hainan92-84 (*E. arundinaceus*) exhibited the most extreme values at the opposite end of the observed variation (C_i_ = 102 μmol mol^−1^ and TE_i_ = 164 μmol CO_2_ mol^−1^ H_2_O).

**Table 2.** Summary statistics from analysis of variance, and estimates of H_b_ on the basis of all data or a single measurement

Statistic	*A* (μmol m^−2^ s^−1^)	g_s_ (mol m^−2^ s^−1^)	C_i_ (μL L^−1^)	TE_i_ (*A*/g_s_) (μmol mol^−1^)	TEg_s_ (μmol mol^−1^)	TEpc (μmol mol^−1^)
GCV (%)	25.3	27.4	8.6	5.3	4.0	2.9
σ_clones_^2^	19.1^***^	0.00122^***^	132^***^	55.8^***^	32.1^***^	16.9^***^
σ_clone × dates_^2^	4.81^**^	0.00029^**^	5.1 ns	7.9 ns	4.92^**^	0.52 ns
σ_error_^2^	36.3	0.00278	1198	470	63.0	183.1
H_b_ (all data basis)	0.96	0.96	0.87	0.88	0.94	0.91
H_b_ (single measure basis)	0.32	0.29	0.10	0.10	0.21	0.14

ns = not significant; **P < 0.05; ***P < 0.001. Analysis was carried out as described in the “Materials and methods”.

Measurements of *A* and g_s_ had much higher GCVs compared with C_i_ and TE (Table 2). Variation due to clone × date interaction was relatively small for all measurements compared with genotypic variance (Table 2), indicating that clones responded reasonably similarly to each other across the range of conditions experienced during the course of the experiment. Error variance was large relative to genotypic variance (about 2-fold larger for *A* and g_s_ and 8-fold larger for TE) but H_b_ based on all data was high (>0.8) for all measurements (Table 2), due to the large number (≥63) of observations made on each clone (Fig. 2). This demonstrated accurate discrimination among the clones for this number of measurements (i.e. >85% of variation in observed genotype means attributed to genotypic effects).

Expected values of H_b_ on the basis of different numbers of measurements can be used to help develop optimal strategies for future germplasm screening experiments, and these are indicated in Fig. 2. In any particular screening effort with a finite set of resources available for measurements (i.e. assuming there is a particular maximum number of measurements that may be made), there exists a compromise between the desire to obtain a high accuracy of characterizing each individual genotype through more measurements and increasing numbers of genotypes which may be screened. From a breeding programme perspective, the aim is usually to maximize the gain from selection (Equation 8), and this can be calculated for different experimental configurations (e.g. genotype number × number of measurements per genotype) (Falconer and Mackay, 1996). Predicted gain from selection is given in Table 3 for different options for screening clones and assuming that a fixed number of 3000 measurements can be made.

**Table 3.** Predicted gain in TE_i_ from selection of the top 10 clones

Number of measurements per clone	Number of clones able to be screened	Gain in TE_i_ from selecting the top ranked 10 clones for TE_i_ (μmol mol^−1^)
1	3000	7.4
2	1500	9.2
4	750	11.1
8	375	12.1
16	188	12.3
32	94	11.7
64	47	9.2

Predictions are for different combinations of measurements per genotypes × number of genotypes, and assuming a fixed number of 3000 measurements are made.

### Relationships between gas exchange parameters

**Fig. 3.**
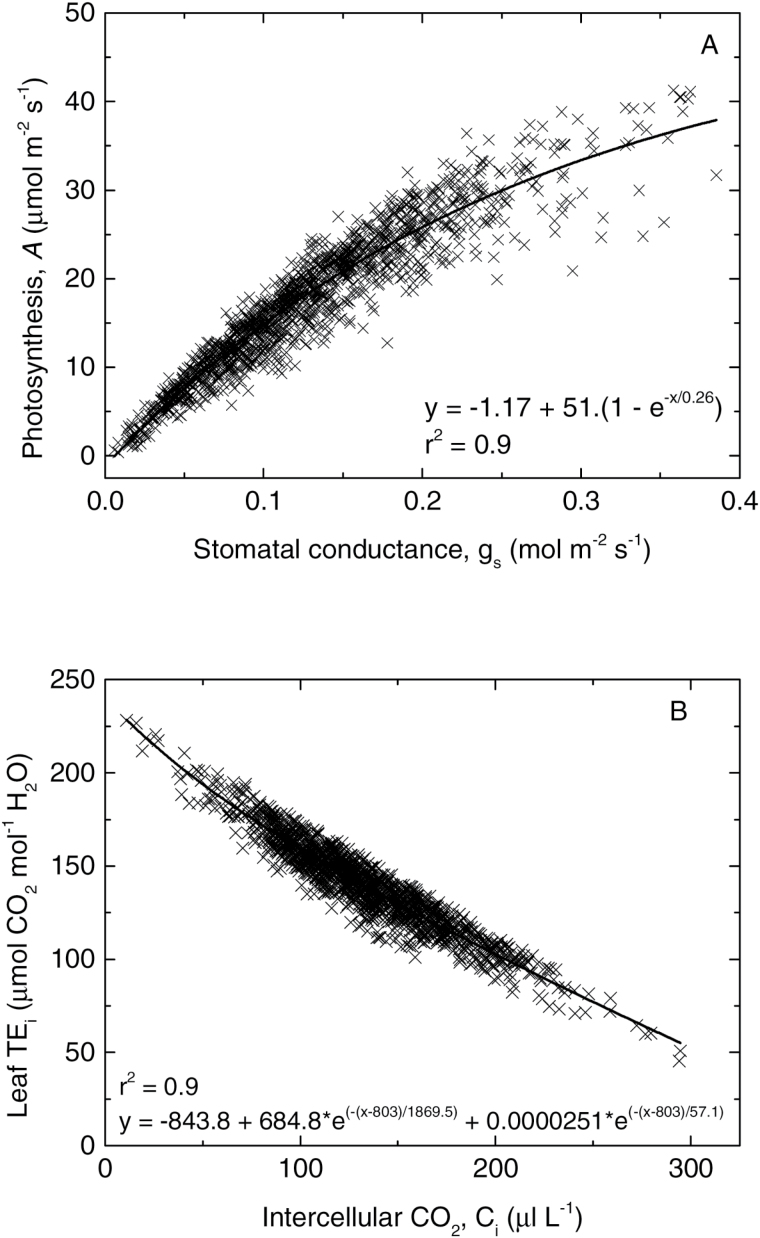
Relationship between leaf gas exchange parameters. (**A**) The relationship between *A* and g_s_ and (**B**) the relationship between TE_i_ and C_i_, for all data collected. The solid line is a best fit exponential equation for all data points.

There was a strong positive correlation between *A* and g_s_ (Table 4). As expected, this relationship was curvilinear (Fig. 3A) and consequently resulted in low values of g_s_ associated with higher TE_i_ (*A*/g_s_) (as reported elsewhere), and a negative relationship between g_s_ and TE_i_ (Table 4). As expected, there was also a strong negative relationship between C_i_ and TE_i_ (Fig. 3B, Table 4).

**Table 4.** Correlations among leaf gas exchange parameters

	*A*	g_s_	TE_i_	C_i_	TEg_s_	TEpc
*A*	1.00					
g_s_	0.94	1.00				
TE_i_	−0.13	−0.41	1.00			
C_i_	−0.18	0.12	−0.95	1.00		
TEg_s_	−0.63	−0.73	0.40	−0.19	1.00	
TEpc	0.23	−0.02	0.85	−0.92	−0.14	1.00

Pearson’s correlation coefficients for each measurement are shown based on all data collected. All correlations are statistically significant (*P* < 0.001, *n* = 1325).

### Variation in components of leaf TE_i_

**Fig. 4.**
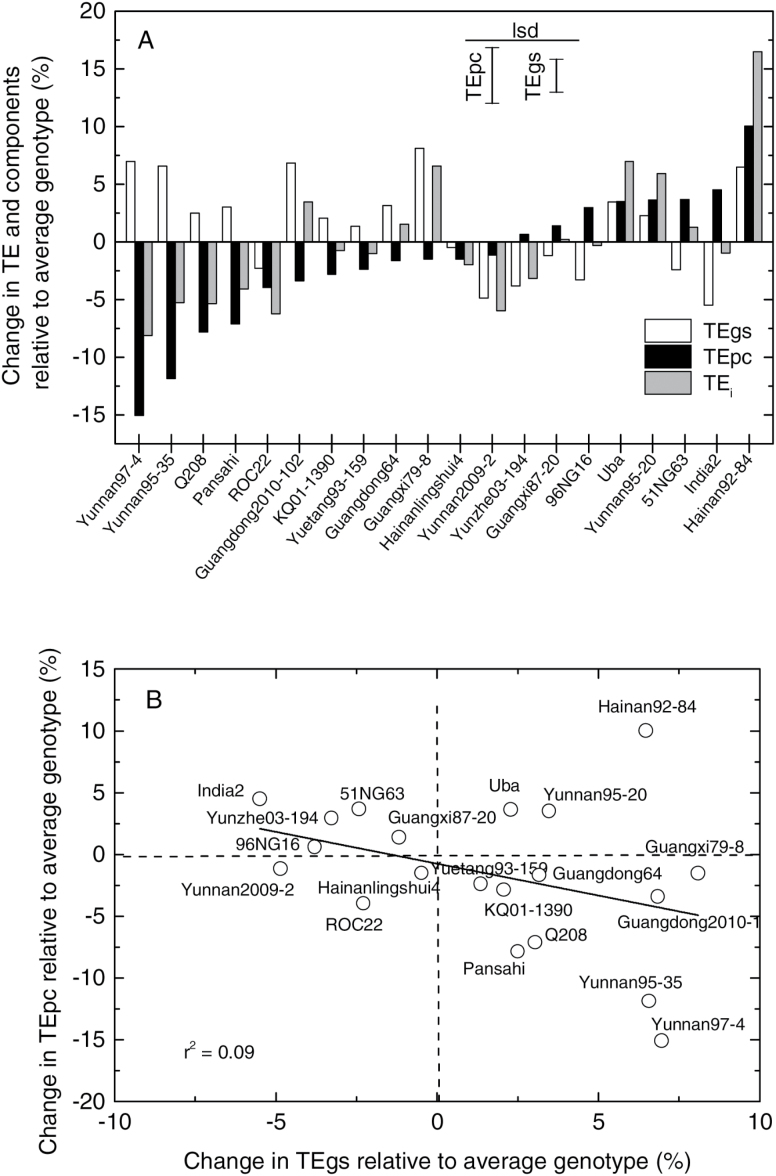
Relationship between components of TE_i_. (**A**) Changes in TEpc and TEg_s_ for each genotype expressed relative to the average of all genotypes. (**B**) Relationship between genotype performance for TEpc and TEg_s_.

Variation in both TEg_s_ and TEpc contributed to overall variation in TE_i_ among the genotypes (Table 2, Fig. 4). Genotypic variation in TEg_s_ was slightly larger than in TEpc (Table 2), but overall TE_i_ was more strongly correlated with TEpc than TEg_s_ (Table 4). TEpc and TEg_s_ were only weakly related (Table 4, Fig. 4B). The TE_i_ and TEpc levels of the two commercial cultivars (Q208 and ROC22) included in this study were clearly exceeded by some of the other wild clones (Fig. 4). The genotype with the highest observed TE_i_ (Hainan92-84, *E. arundinaceus*) had a higher than average TEg_s_ and very high TEpc (Fig. 4). Two other clones, Yunnan95-20 (*E. rockii*) and Uba (*S. sinense*), also showed high TEpc associated with high TE_i_ (Fig. 4).

## Discussion

The results from this study indicate that differences in photosynthetic capacity contribute to a significant portion of the variation in TE_i_, rather than only differences attributed to stomatal conductance. This is important because it means that, with concerted breeding and selection, high levels of TE_i_ should be attainable without necessarily low levels of conductance, and therefore without necessarily sacrificing high rates of photosynthesis and productivity (Blum, 2009).

The set of clones included in this experiment was a small sample of those available in the main Chinese germplasm collections, but the results obtained in this study are encouraging in supporting further surveys of germplasm for high TE. Several wild species, most notably the *E. arundinaceus* clone Hainan92-84, exhibited high TEpc compared with popular Australian (Q208) and Chinese (ROC22) commercial cultivars included in this experiment. *E. arundinaceus* has been of interest among sugarcane breeders for its reputed ability to produce high biomass in dry environments (Jackson and Henry, 2011), although there appears to be little or no substantive data supporting this reputation. It would seem likely that other, possibly more extreme, levels of TEpc may be observed in larger-scale screening of germplasm. Genotypic variances found for *A*, g_s_, and C_i_ in this study were similar in magnitude to those found in a study of sugarcane-related germplasm by Jackson *et al.* (2016), but partitioning of TE_i_ into components due to variation in conductance or photosynthetic capacity was not reported in that study.

The data obtained here may be used to design optimal methods for further larger-scale screening of germplasm collections and progeny derived from any breeding efforts. Results obtained from this study suggested about 16 measurements per clone would represent a near-optimal approach in further screening efforts, balancing the competing benefits of screening a large number of clones versus a large number of measurements to decrease experimental error variance. The small magnitude of clone × date interaction for TE_i_ and TEpc is a positive result in that it suggests that relative ranking of clones for these traits was reasonably consistent under different weather conditions (at least within the range of conditions experienced in this study).

The experiment was conducted under conditions of high water availability with minimal or no water stress. Responses measured under these conditions are relevant to water-limited production environments because in many field crops a high proportion of total water use across a whole cropping growth cycle usually occurs on days when water uptake is largely meeting crop demand (Ritchie, 1973), and this is also expected to be the case for most commercial sugarcane production environments. In related work, using a modified version of the APSIM sugarcane model (Inman-Bamber *et al.*, 2016), we estimated that >70% of water is used in typical rain-fed sugarcane production environments in Australia on days when the crop is non-stressed (data not shown). In other words, in commercial production environments a small proportion of transpiration is expected to take place on days when there is moderate-to-severe water stress, because of stomatal closure. Therefore, in relation to impacting on final yield, increasing TE_i_ on days when there is no or mild stress will be proportionally more important than the same increase in TE_i_ during times of moderate or severe stress. For any given growth rate, genotypes which have a higher TE_i_ under non-stress conditions will use water at a slower rate and therefore have a delayed onset of water stress and greater total biomass production at harvest in environments in which periods of water stress occur.

Reduced g_s_ (either generally or in response to any environmental factors) will act to reduce C_i_ levels and hence increase intrinsic TE (via equation 5). This may contribute to the well-known curvilinear relationship between *A* and g_s_ (Fig. 3A), leading to a negative relationship between TE_i_ and g_s_, which was also clearly observed in this study (Table 4). The possibility of negative genotypic correlations between conductance and TE_i_ has been highlighted as a major complication and limitation in using TE as a selection criterion (e.g. Blum, 2005, 2009; Condon *et al.*, 2004). Genotypes with high TE_i_ arising through reduced conductance may have reduced total transpiration during the entire crop growth cycle, leading to reduced biomass and yield (through equation 1). However, the partitioning of variation of TE_i_ into that expected due to the general curvilinear relationship observed between *A* and g_s_ and deviations from this relationship attributed to differences in photosynthetic capacity should allow for different weightings to be placed on these two components in breeding and selection. Variation in TE_i_ predominately arising from high photosynthetic capacity would be expected to be a more prized target than that due to low conductance, which is likely to be associated with low productivity except perhaps under the most extreme water-limited environments. This includes clones Hainan92-84 (*E. arundinaceus*) and Uba (*S. sinense*), which both exhibited high TE_i_ associated with high TEpc in this study (Fig. 4).

There were two minor differences in the methodology used in this study to partition genotypic TE_i_ effects than in that previously used by Gilbert *et al.* (2011). First, in our analysis, the mean response of all genotypes was used as the reference response rather than that of an individual reference genotype. Second, TEpc for each genotype was estimated based on its typical operating g_s_ value rather than the mean g_s_ of the reference genotype. Our methodology gave estimates for both TEpc and TEg_s_, which correlated strongly with those of Gilbert *et al.* (2011) (r = 0.81 for TEpc and r = 0.8 for TEg_s_) across genotypes. However, one possible advantage of our approach for future screening of germplasm is that it does not require estimation of the curvilinear *A* versus g_s_ function, which avoids the requirement of a large number of data points for each genotype to accurately establish this relationship. The approach used in this report requires only that estimates of the typical operating levels of *A* and g_s_ be made for each genotype, which, assuming similar conditions to those experienced in this study are encountered and based on suggestions above, may require about 16 measurements to maximize selection gains. This should facilitate faster screening of larger genotypic populations than if the *A* versus g_s_ relationship needs to be accurately characterized for each genotype.

Overall, the results suggest that heritable genotypic variation in TEi measured on individual leaves exists within sugarcane-related germplasm and could be targeted in breeding programmes. However, one important reservation in pursuing this strategy at this stage is the uncertainty about the extent to which genotypic variation in TEi measured in plants in pots will translate to whole-plant or crop-level TE (TE_P_) in the field. According to (Farquhar *et al.*, 1989), TE_P_ can be expressed as:

$${\text{TE}}_{P}=\frac{\text{Biomass}}{\text{Water use}}=\frac{C_{a}(1-C_{i}/C_{a})(1-\varphi_{c})}{1.6(e_{i}-e_{a})(1+\varphi_{w})}=\frac{\left(1-\varphi_{c}\right)}{1.6(e_{i}-e_{a})(1+\varphi_{w})}{\text{TE}}_{i}$$ (9)

where ϕ_c_ is the proportion of carbon lost from the shoot at night or from non-photosynthetic tissues such as roots, during the day and night; and ϕ_w_ is the proportion of unproductive water loss such as cuticular water losses or nocturnal stomatal water losses. This expression highlights the importance of whole-plant biomass partitioning, a trait that can vary genetically and exert significant mediator effect between leaf-level and crop-level TE. At the leaf level, equations 4 and 9 also highlight environmental interactions with TE_i_. In particular, two mechanisms may act to reduce the correlation between measurements made in experiments, such as those reported here, and genotypic effects in field production environments. First, genotypic variation in conductance sensitivity to increasing vapour pressure deficits, which has been examined in a range of crops (e.g. Fletcher *et al.*, 2007; Sinclair *et al.*, 2008; Sadok and Sinclair, 2009; Devi *et al.*, 2010; Gholipoor *et al.*, 2010; Gilbert *et al.*, 2011; Belko *et al.*, 2012; Belko *et al.*, 2013; Yang *et al.*, 2012; Gholipoor *et al.*, 2013), may provide an additional source of genotypic variation in TE in addition to TEi. Second, any de-coupling between variation in stomatal conductance and transpiration at a crop canopy scale (Jarvis and McNaughton, 1986) will act to moderate differences occurring at the leaf level. Jackson *et al.* (2016) found that leaf-level TE was correlated with whole-plant TE but the plants were not grown to produce a full canopy. In a dense canopy with unstirred and humid air, stomata can exert little influence over transpiration above very low levels of conductance (e.g. Bange, 1953; Jarvis and McNaughton, 1986). By contrast, in leaves in sparse canopies and subjected to high air movement, stomata exert a high level of control over water loss. In young sugarcane crops in spring prior to wet seasons and when water availability often greatly reduces growth, it may be expected that there would be strong coupling between stomata and whole-canopy transpiration. However, further studies need to be done to examine the extent to which genotypic variation in TE measured at the leaf level in pots translates to whole-crop differences in the field.

## Conclusions

This study was undertaken as part of a larger project aiming at exploring the usefulness of screening for physiological traits in large-scale breeding programmes. We found significant genotypic variation in leaf TEi that was due to the joint contribution of variation in g_s_ and photosynthetic capacity at normal conductance operating levels. We identified a number of genotypes possessing high TEi as a result of high photosynthetic capacity, which could have significant breeding value because this measure should not be negatively associated with the reduced growth rates caused by lower g_s_. We also established that a small number of simple leaf gas exchange measurements is sufficient to efficiently screen TEi and apportion the variation related to the photosynthetic component. For practical applications in breeding, investigations should now turn to establishing narrow sense heritability of these traits as well as the leaf-to-field translational potential of the TE_i_ trait reported in this study.

## Abbreviations:

A: CO_2_ assimilation rate
C_a_: ambient CO_2_ concentration
C_i_: intercellular CO_2_ concentration
E: H_2_O vapour transpiration
e_a_: ambient H_2_O vapour pressures
e_i_: H_2_O vapour pressures inside the leaf
GCV: genotypic coefficient of variation
g_s_: stomatal conductance
gs_CO_2_: stomatal conductance for CO_2_
gs_H_2_O: stomatal conductance for H_2_O vapour
H_b_: broad sense heritability
TE: transpiration efficiency
TEg_s_: transpiration efficiency estimated from variation in stomatal conductance
TE_i_: intrinsic transpiration efficiency
TE_L_: leaf-level transpiration efficiency
TE_P_: transpiration efficiency of whole plant
TEpc: transpiration efficiency estimated from variation in photosynthetic capacity
ϕ_c_: proportion of consumptive carbon lost
ϕ_w_: proportion of unproductive water loss.
